# Avaliação Prognóstica da Reserva de Fluxo Fracionada em Diferentes Estratos nos Pacientes com Doença Arterial Coronariana

**DOI:** 10.36660/abc.20211051

**Published:** 2023-06-07

**Authors:** Denise Pellegrini, Paulo R. A. Caramori, Ricardo Czarnobai Soccol, Ricardo Lasevitch, Gustavo Luís Agostini, Alice Dussin, Flavio Vinicius Costa Ferreira, Mario Bernardes Wagner, Luiz Carlos Bodanese

**Affiliations:** 1 Pontifícia Universidade Católica do Rio Grande do Sul Porto Alegre RS Brasil Pontifícia Universidade Católica do Rio Grande do Sul, Porto Alegre, RS – Brasil; 2 Hospital São Lucas PUCRS Porto Alegre RS Brasil Hospital São Lucas da PUCRS, Porto Alegre, RS – Brasil

**Keywords:** Reserva de Fluxo Fracionada, Desfechos, Isquemia

## Abstract

**Fundamento:**

Existem dados limitados sobre a evolução clínica de lesões coronarianas não tratadas de acordo com sua gravidade funcional no mundo real.

**Objetivo:**

Este estudo teve como objetivo avaliar os resultados clínicos de até 5 anos em pacientes com lesões revascularizadas com reserva de fluxo fracionada (FFR) ≤ 0,8 e em pacientes com lesões não revascularizadas com FFR > 0,8.

**Métodos:**

A avaliação pelo FFR foi realizada em 218 pacientes seguidos por até 5 anos. Os participantes foram classificados com base na FFR no grupo isquêmico (≤ 0,8, grupo intervenção, n = 55), no grupo FFR normal-baixa (> 0,8-0,9, n = 91) e no grupo FFR normal-alta (> 0,9, n = 72). O desfecho primário foram eventos cardíacos adversos maiores (ECAMs), um composto de morte, infarto do miocárdio e necessidade de nova revascularização. O nível de significância adotado neste estudo foi alfa = 0,05; deste modo, resultados com valores de p < 0,05 foram considerados estatisticamente significativos.

**Resultados:**

A maioria dos participantes era do sexo masculino (62,8%) com média de idade de 64,1 anos. Diabetes estava presente em 27%. À angiografia coronariana, a gravidade da estenose avaliada foi de 62%, 56,4% e 54,3% nos grupos isquêmico, FFR normal-baixa e FFR normal-alta, respectivamente (p < 0,05). O período médio de acompanhamento foi de 3,5 anos. A incidência ECAM foi de 25,5%, 13,2% e 11,1%, respectivamente (p = 0,037). Não houve diferença na incidência de ECAM entre os grupos FFR normal-baixa e FFR normal-alta (p = NS).

**Conclusão:**

Pacientes com FFR indicativa de isquemia apresentaram piores desfechos quando comparados aos dos grupos não isquêmicos. Entre os grupos que apresentaram valores de FFR considerados normal-baixo e normal-alto, não houve diferença na incidência de eventos. Há necessidade de estudos de longo prazo e com grande número de pacientes para melhor avaliar os desfechos cardiovasculares em pacientes portadores de estenose coronariana moderada com valores de FFR entre 0,8 e 1,0.

**Figura Central:**
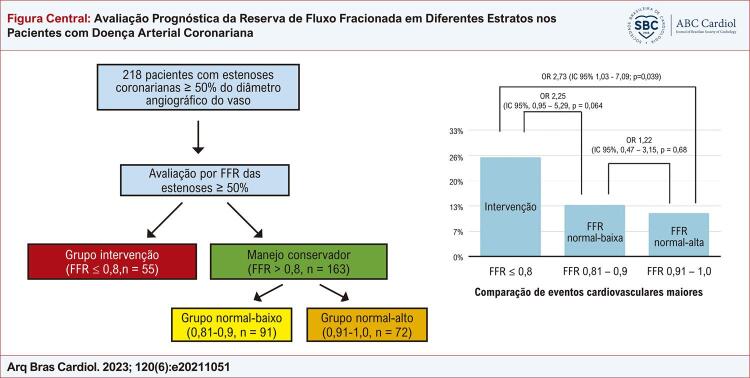
: Avaliação Prognóstica da Reserva de Fluxo Fracionada em Diferentes Estratos nos Pacientes com Doença Arterial Coronariana

FFR: reserva de fluxo fracionada; OR: razão de chances (de odds ratio); IC: intervalo de confiança.

## Introdução

A avaliação fisiológica da doença arterial coronariana (DAC) tornou-se um dos pilares na decisão da revascularização do miocárdio.^1^ A gravidade anatômica das lesões coronarianas está associada a eventos adversos.^2 - 4^

A reserva de fluxo fracionada (FFR) surgiu como a ferramenta de referência para a avaliação da gravidade funcional das lesões coronarianas. Sua significância no tratamento da DAC foi ressaltada nos últimos anos pela observação de que a revascularização coronariana, de acordo com a significância funcional da lesão, está associada a desfechos clínicos melhores a longo prazo.^4 - 7^ Existem dados já publicados quanto aos resultados clínicos das lesões coronarianas não revascularizadas de acordo com a gravidade funcional,^8 , 9^ e acredita-se que os desfechos clínicos sejam diferentes de acordo com diferentes estratos da FFR. Contudo, não há uniformidade nos estudos quanto à estratificação dos valores da FFR, podendo os desfechos, desta forma, serem diferentes. Além disso, os fatores clínicos que estão associados a resultados clínicos adversos em lesões não revascularizadas também diferem entre os estudos. Descrever a evolução e identificar os fatores prognósticos de lesões não revascularizadas têm grande relevância na prática clínica. Portanto, este estudo teve como objetivos 1) avaliar os resultados clínicos de 5 anos das lesões não tratadas de acordo com a gravidade funcional e 2) definir os fatores associados aos resultados adversos em lesões não tratadas. Devido à progressão da doença aterosclerótica, o risco de eventos aumenta gradualmente com a redução dos valores da FFR.^10^ Assim, os pacientes deixados sem revascularização com uma FFR “baixa” (ou seja, de 0,80 a 0,90) poderiam estar em maior risco de isquemia ou complicações do que aqueles com valores mais altos (> 0,90).

O presente estudo pretendeu avaliar a incidência de desfechos em pacientes com FFR em diferentes estratos após um período de acompanhamento de até 5 anos.

## Material e métodos

### Desenho do estudo

Tratou-se de um estudo observacional de coorte histórica. Os dados foram coletados prospectivamente e armazenados no banco de dados da unidade de cardiologia intervencionista.

#### Desfechos do estudo

1. Composto, eventos cardiovasculares maiores (óbito, infarto do miocárdio e necessidade de nova revascularização);
2. Óbito;
3. Infarto do miocárdio;
4. Acidente vascular cerebral isquêmico;
5. Necessidade de nova revascularização; e
6. Revascularização do vaso-alvo.

## População em estudo

A população deste estudo foi composta por pacientes encaminhados à Unidade de Hemodinâmica e Cardiologia Intervencionista, no período de janeiro de 2013 a setembro de 2018, submetidos à avaliação da FFR de ao menos uma lesão coronariana com estenose de pelo menos 50%.

## Critérios de inclusão

1. Pacientes com indicação de avaliação funcional da lesão coronariana; e
2. Estenoses coronarianas ≥ de 50% do diâmetro do vaso pela estimativa visual da angiografia.

Critérios de exclusão

1. Lesão do tronco da coronária esquerda;
2. Choque cardiogênico;
3. Cirurgia de revascularização do miocárdio prévia;
4. Artérias coronárias extremamente tortuosas e/ou calcificadas;
5. Expectativa de vida inferior a 2 anos; e
6. Pacientes grávidas.

## Protocolo do estudo

Foram registrados dados angiográficos e não angiográficos.

### Angiografia coronariana

A angiografia coronariana foi realizada com cateteres diagnósticos 5 F ou 6 F, através de acesso femoral ou radial. Foi realizada a administração seletiva intracoronária de 200 mcg de nitroglicerina antes do início da angiografia das coronárias esquerda e direita.^5 , 6^

Múltiplas imagens cineangiográficas das coronárias esquerda e direita foram obtidas, em múltiplas projeções nas angulações oblíqua anterior esquerda ou direita, craniais ou caudais quando necessário.^7 , 10^

### Reserva de fluxo fracionada (FFR)

Um cateter-guia 6 F de rotina foi utilizado para cateterizar seletivamente a coronária para realização da FFR, com atenção ao alinhamento coaxial com o óstio da coronária para evitar o *damping* da curva de pressão. Após, um fio-guia de 0,014’ com monitorização de pressão foi calibrado à pressão atmosférica e avançado até a extremidade do cateter-guia. A seguir, foi realizada a equalização com a pressão aórtica (Pa) do cateter-guia. O fio-guia foi, então, avançado a parte distal do vaso para registrar a pressão coronária distal (Pd), garantindo que o sensor de pressão esteja localizado além da lesão a ser avaliada. A FFR das lesões das coronárias esquerda e direita foram registradas durante a hiperemia máxima induzida com adenosina endovenosa em um acesso periférico de bom calibre na dose de 140 µm/kg/min, durante 3 a 5 minutos.^1 , 8 , 11^

O valor da FFR foi automaticamente calculado como o gradiente de pressão (Pa/Pd) mais baixo atingido durante a hiperemia máxima. Um valor de FFR menor ou igual a 0,8 foi definido como hemodinamicamente significativo, ou seja, anormal de acordo com as evidências atuais; assim, foi indicada a revascularização.^8 , 9 , 12^ Pacientes com lesões com FFR maior do que 0,8 foram mantidos em acompanhamento clínico.

Os participantes foram classificados com base na FFR no grupo isquêmico (≤ 0,8, grupo intervenção, n = 55), no grupo FFR normal-baixa (> 0,8-0,9, n = 91) e no grupo FFR normal-alta (> 0,9, n = 72). Os pacientes do grupo isquêmico foram revascularizados com *stents* farmacológicos.

### Parâmetros angiográficos

A análise angiográfica quantitativa (AAQ) da lesão que foi funcionalmente avaliada foi obtida utilizando o Cardiac Viewer (™Philips). Pacientes com pelo menos 50% de estenose coronariana (por análise visual) em pelo menos dois vasos foram considerados como portadores de doença multivascular.

### Acompanhamento e desfechos clínicos

Os pacientes foram seguidos por até 5 anos após o procedimento. O acompanhamento foi realizado prospectivamente através de consulta telefônica. Os desfechos clínicos foram definidos como morte, infarto agudo do miocárdio, nova revascularização do vaso-alvo, nova hospitalização e acidente vascular cerebral. A revascularização do vaso-alvo é definida como qualquer revascularização percutânea ou cirúrgica no vaso previamente tratado devido à reestenose ou outra complicação relacionada à lesão.

## Tamanho da amostra

Para detectar uma diferença de eventos cardiovasculares maiores de 12% nos grupos FFR normal-baixa (0,8-0,9) e FFR normal-alta (> 0,9) e de 30% no grupo isquêmico em uma razão de 3 para 1, com um poder estatístico de 80% em um nível de significância bicaudal de 5%, foi estimado que seriam necessários 150 pacientes nos grupos FFR normal-baixa e FFR normal-alta e 50 no grupo isquêmico; ou seja, um total de 200 pacientes.

## Análise estatística

Dados quantitativos foram descritos por média e desvio-padrão. Com base no teorema central do limite, não foram utilizados testes estatísticos para verificação da normalidade dos dados quantitativos. Dados categóricos foram expressos por contagem e percentuais. A comparação das médias foi realizada por análise de variância (ANOVA) one-way, e a comparação dos eventos categóricos foi realizada pelo teste do qui-quadrado ou exato de Fisher. Quando necessário, foi utilizado o procedimento de *post-hoc* de Tukey para média e o ajuste de Benjamini-Hochberg para situações categóricas. Inicialmente foram obtidas estimativas de razão de chances (OR, de *odds ratio* ) e seus respectivos intervalos de confiança (IC) de 95% na análise univariada com significância baseada no teste de qui-quadrado. A seguir, modelos de regressão logística, como análise multivariada, foram utilizados para o cálculo de ORs ajustadas para potenciais efeitos confundidores. O nível de significância (erro tipo I bicaudal) adotado neste estudo foi de alfa = 0,05; deste modo, resultados com valor p < 0,05 foram considerados estatisticamente significativos. Os cálculos foram efetuados com auxílio do programa IBM SPSS, versão 25.0.

## Resultados

Os participantes foram divididos de acordo com o valor de FFR em grupos isquêmico (FFR ≤ 0,80, ou intervenção, n = 55), FFR normal-baixa (> 0,8-0,9, não revascularizados, n = 91) e FFR normal-alta (> 0,9, não revascularizados, n = 72). No período de janeiro de 2013 a setembro de 2018, foram incluídos 241 pacientes; destes, 218 completaram acompanhamento (90,4%). O período médio de acompanhamento foi de 3,5 anos. A média de idade dos indivíduos foi de 64,1 anos. A maioria dos pacientes era do sexo masculino (62,8%), e diabetes melito estava presente em 27% dos pacientes. As demais características clínicas estão apresentadas na Tabela 1 . Não houve diferença entre os grupos quanto a maior parte das características clínicas basais, excetuando-se a apresentação clínica ( Tabela 1 ).

**Tabela 1:** – Dados demográficos

Características clínicas	Grupo isquêmico n = 55	Grupo normal-baixo n = 81	Grupo normal-alto n = 53	Valor de p
Idade (anos)	63,2±10,3	65,1±10,4	64,2±10,2	0,67
Gênero masculino	37 (67,3)	58 (63,7)	42 (58,3)	0,57
Hipertensão, n (%)	39 (70,9)	51 (56,0)	49 (68,1)	0,12
Dislipidemia, n (%)	26 (47,3)	35 (38,5)	36 (50,0)	0,30
Tabagismo, n (%)	14 (27,0)	20 (21,9)	23 (31,9)	0,47
Diabetes, n (%)	17 (30,9)	23 (25,3)	19 (25,0)	0,70
Infarto prévio, n (%)	12 (21,8)	10 (11,0)	14 (19,4)	0,16
Angioplastia coronariana prévia, n (%)	21 (38,2)	25 (27,5)	24 (33,3)	0,24
Cirurgia de revascularização prévia, n (%)	1 (1,8)	5 (5,5)	6 (8,3)	0,28
**Apresentação inicial, n (%)**				
Angina crônica	41 (74,5)	81 (89,0)	53 (73,6)	0,02
Angina instável	9 (16,4)	7 (7,7)	10 (13,9)	0,24
Infarto sem supra do segmento ST	5 (9,1)	3 (3,3)	8 (11,1)	0,13
Infarto com supra do segmento ST	0 (0,0)	0 (0,0)	1 (1,4)	0,36
**Medicações, n (%)**				
AAS	43 (78,2)	67 ( 73,6)	52 (74,3)	0,64
Clopidogrel	30 (54,5)	49 (53,8)	36 (51,4)	0,72
Estatina	42 (76,4)	71(78)	52 (74,3)	0,75
Betabloqueador	33 (60,0)	56 (61,5)	39 (55,7)	0,59
Diurético	12 (21,8)	22 (24,2)	20 (28,6)	0,39
IECA	8 (14,5)	19 (20,9)	22 (31,4)	0,07
BRA	15 (23,7)	27 (29,7)	18 (25,7)	0,75
Hipoglicemiante oral	15 (27,3)	17 (18,7)	17 (24,3)	0,76

*AAS: ácido acetilsalicílico; IECA: inibidor da enzima conversora de angiotensina; BRA: bloqueadores dos receptores da angiotensina.*

Quanto às características angiográficas, o vaso avaliado com maior frequência foi a artéria descendente anterior no grupo isquêmico, seguida pelo grupo normal-baixo ( Tabela 2 ). O grau de estenose angiográfica também diferiu entre os grupos, sendo de maior gravidade no grupo isquêmico ( Tabela 2 ).

**Tabela 2:** – Dados angiográficos

Dados angiográficos	Grupo isquêmico n = 55	Grupo normal-baixo n = 91	Grupo normal-alto n = 72	Valor de p
Vaso avaliado				< 0,001
Artéria descendente anterior, n (%)	47 (85,5)	60 (65,9)	31 (43,1)	*
Outros vasos além da artéria descendente anterior, n (%)	8 (14,5)	31 (34,1)	41 (56,9)	
Estenose angiográfica (%)	62,0±7,7	56,5±6,2	54,0±8,3	< 0,001
FFR	0,75±0,03	0,85±0,02	0,94±0,03	< 0,001

* p = < 0,001 para frequência de avaliação da artéria descendente anterior entro os grupos normal-baixo e normal-alto. FFR: reserva de fluxo fracionada.

**Figura 1:**
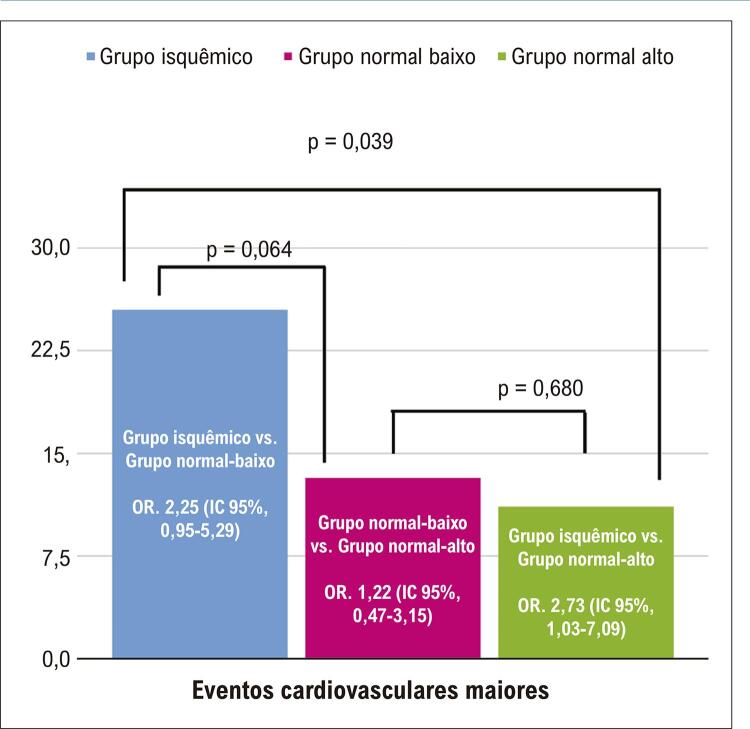
– Comparação de eventos cardíacos adversos maiores (ECAMs) entre os grupos.

O desfecho primário (eventos cardíacos adversos maiores [ECAMs]), combinado de morte, infarto, necessidade de nova revascularização, nova hospitalização e acidente vascular cerebral, foi significativamente diferente entre os grupos, ocorrendo em 25,5% no grupo isquêmico, em 13,2% no grupo FFR normal-baixa e em 11,1% no grupo FFR normal-alta (p = 0,037) ( Tabela 3 ). A OR para a ocorrência de ECAM do grupo isquêmico para o grupo FFR normal-alta foi de 2,73 (IC 95% 1,03-7,09; p = 0,039). A OR para ocorrência de ECAM do grupo isquêmico para o grupo FFR normal-baixa foi de 2,25 (IC 95% 0,95-5,29; p = 0,064), e a OR para ocorrência de ECAM do grupo FFR normal-baixa para o grupo FFR normal-alta foi de 1,22 (IC 95% 0,47-3,15; p = 0,680) ( Figura 1 ).

**Tabela 3:** – Desfechos

Desfechos, n (%)	Grupo isquêmico n = 55	Grupo normal-baixo n = 91	Grupo normal-alto n = 72	Valor de p
Desfecho combinado	14 (25,5)	12 (13,2)	8 (11,1)	0,037
Óbito	2 (3,6)	6 (6,6)	3 (4,2)	0,67
Infarto agudo do miocárdio	1 (1,8)	3 (3,3)	1 (1,4)	0,70
Acidente vascular cerebral	1 (1,8)	0 (0,0)	1 (1,4)	0,46
Necessidade de nova angioplastia	12 (21,8)	5 (5,5)	5 (7,1)	0,04
Revascularização do vaso-alvo	6 (10,9)	3 (3,3)	3 (4,3)	0,12
Necessidade de nova internação	12 (23,6)	6 (6,6)	6 (8,6)	0,05
Necessidade de cateterismo cardíaco	15 (27,3)	7 (7,7)	10 (14,3)	0,05

A mortalidade foi 3,6%, 6,6% e 4,2% nos grupos isquêmico, normal-baixo e normal-alto, respectivamente (p = 0,67). Infarto do miocárdio ocorreu em um paciente do grupo isquêmico, em três pacientes do grupo normal-baixo e em um paciente do grupo normal-alto (p = 0,70). Houve maior necessidade de nova angioplastia durante o seguimento clínico em 21,8% dos pacientes no grupo isquêmico, significativamente maior do que os 5,5% no grupo normal-baixo e do que os 7,1% no grupo normal-alto (p = 0,04) ( Tabela 3 ). Acidente vascular cerebral ocorreu em um paciente no grupo isquêmico e FFR normal-alta. Não houve ocorrência de acidente vascular cerebral no grupo FFR normal-baixa. Maior necessidade de internação hospitalar ocorreu no grupo isquêmico, em 23% dos indivíduos, significativamente maior do que nos grupos FFR normal-baixa e FFR normal-alta, em que foram de 6,6% e 8,6%, respectivamente (p = 0,01).

Na análise de subgrupo, quando são avaliados os pacientes com síndrome coronariana crônica – 175 dos 218 pacientes (80,2%), ou seja, a maioria dos participantes do estudo –, observa-se diferença significativa na ocorrência de ECAM entre os grupos. Os pacientes com FFR ≤ 0,8 apresentaram uma ocorrência de eventos de 29,3%, o estrato FFR normal-baixa apresentou ocorrência de 11,1%, e o estrato FFR normal-alta, de 7,5% ( Tabela 4 ). A ocorrência de ECAM foi diferente provavelmente às custas de maior necessidade de nova revascularização no grupo isquêmico. Entre os grupos FFR normal-baixa e FFR normal-alta, não houve diferença significativa quanto à ocorrência de desfechos (p = 0,56).

**Tabela 4:** – Desfechos no subgrupo dos pacientes com doença coronariana crônica

Desfechos, n (%)	Grupo isquêmico n = 41	Grupo normal-baixo n = 81	Grupo normal-alto n = 53	Valor de p
Desfecho combinado	12 (29,3)	9 (11,1)	4 (7,5)	0,004
Óbito	0	0	2 (3,8)	0,07
Infarto agudo do miocárdio	1 (2,4)	2 (2,5)	0	0,34
Acidente vascular cerebral	1 (2,4)	0	1 (2,0)	0,91
Necessidade de nova angioplastia	10 (24,4)	4 (4,9)	1 (2,0)	< 0,001
Revascularização do vaso-alvo	2 (1,1)	1 (2,4)	1	0,26
Necessidade de nova internação	11 (26,8)	5 (6,2)	3 (5,9)	0,003
Necessidade de cateterismo cardíaco	11 (26,8)	6 (7,4)	5 (9,8)	0,02

A análise univariada foi realizada para identificar determinantes de ECAM entre todos os participantes. Na análise de risco univariável, observou-se que sexo masculino, idade > 65 anos, hipertensão arterial sistêmica, diabetes melito, dislipidemia, infarto prévio, angioplastia prévia e cirurgia de revascularização miocárdica (CRM) prévia não foram significativamente associados aos eventos cardiovasculares maiores analisados isoladamente. Contudo, o valor de FFR ≤ 0,80 foi preditor de ECAM (OR 2,73, IC 95% 1,05-7,09; p = 0,039; Tabela 5 ).

**Tabela 5:** – Análise univariada para preditores de eventos cardíacos adversos maiores (ECAMs)

Variável	Sem ECAM n = 184	Com ECAM n = 34	OR	IC 95%	Valor de p
Sexo masculino, n (%)	116 (62,7)	22 (64,7)	0,91	0,42-1,96	0,82
Idade > 60 anos, n (%)	123 (66,5)	22 (64,7)	0,92	0,42-1,98	0,84
FFR, n.º/n.º total (%)					
≥ 0,90	64/184 (34,8)	8/34(23,5)	1	-	-
0,81-0,89	79/184 (42,9)	12/34 (35,3)	1,22	0,47-3,15	0,689
≤ 0,80	41/184 (22,3)	14/34 (41,2)	2,73	1,05-7,09	0,039
Hipertensão, n (%)	120 (64,9)	20 (58,8)	0,77	0,37-1,63	0,561
Diabetes melito, n (%)	46 (24,9)	12 (35)	1,64	0,75-3,5	0,20
Dislipidemia, n (%)	83 (44,9)	14 (41,2)	0,86	0,41-1,80	0,69
Infarto do miocárdio prévio, n (%)	30 (16,2)	6 (17,6)	1,1	0,42-2,90	0,83
Angioplastia prévia, n (%)	10 (5,4)	2 (5,9)	1,09	0,2-5,22	0,91
CRM prévia, n (%)	57 (30,8)	12 (38,2)	1,39	0,65-2,96	0,39

*OR: razão de chances (odds ratio); IC: intervalo de confiança; FFR: reserva de fluxo fracionada; CRM: cirurgia de revacularização miocárdica.*

Na análise multivariada, após o ajuste para os fatores sexo, idade, hipertensão e diabetes melito, houve a manutenção da diferença entre os estratos de FFR na ocorrência de ECAM (OR 2,72, IC 95% 1,03-7,14; p = 0,04).

## Discussão

O presente estudo, envolvendo 218 pacientes com DAC acompanhados por até 5 anos e submetidos à avaliação da FFR, mostrou um número maior de ECAM no grupo isquêmico comparado aos grupos FFR normal-baixa e FFR normal-alta, não havendo diferenças entre estes dois últimos.

Os parâmetros de avaliação da severidade anatômica, como diâmetro da estenose, extensão, excentricidade da placa, ângulo e calcificação, podem ser indicativos de complexidade e prognóstico da lesão. Contudo, o prognóstico de um paciente difere significativamente de acordo com a presença ou não de isquemia miocárdica em uma determinada lesão.^5^ Desta forma, há necessidade de superar as limitações da angiografia na avaliação do impacto funcional da lesão coronariana, para a qual a FFR tem sido utilizada como ferramenta de referência na avaliação fisiológica invasiva.^6^

Nos últimos anos, vários estudos relataram de forma consistente os resultados favoráveis da revascularização por angioplastia guiada por FFR na prática clínica. No estudo DEFER, os resultados clínicos após a não intervenção com base na FFR foram excelentes durante o acompanhamento de 15 anos.^8^ Também foi demonstrado que a avaliação de rotina de FFR reduziu significativamente a ocorrência de desfecho clínico composto de morte, infarto não fatal e revascularização em pacientes com doença coronariana multiarterial que foram tratados com *stent* farmacológico no estudo Fractional Flow Reserve versus Angiography for Multivessel Evaluation (FAME 1).^10^ Além disso, a angioplastia guiada por FFR associada à terapia médica otimizada diminuiu a necessidade de revascularização de urgência em comparação com a terapia médica otimizada isolada em pacientes estáveis com lesão coronariana funcionalmente significativa, de acordo com o estudo FAME 2.^13^ Considerando os resultados desses estudos, o tratamento das lesões coronarianas guiado pela avaliação da FFR pode garantir melhores desfechos clínicos.

No presente estudo, o prognóstico em longo prazo de indivíduos com lesões coronarianas isquêmicas revascularizadas foi significativamente pior do que o de pacientes com lesões coronarianas não isquêmicas que não foram revascularizadas, indicando que, no mundo real, mesmo a revascularização com *stents* farmacológicos associada ao manejo clínico convencional não foi capaz de reduzir os desfechos nos pacientes isquêmicos ao ponto de torná-los comparáveis aos desfechos de pacientes livres de isquemia pela FFR.

A maior ocorrência de ECAM no grupo isquêmico se deu às custas de maior necessidade de nova revascularização fundamentalmente de outras lesões. Esse achado indica que pacientes com lesões isquêmicas apresentam pior evolução clínica relacionada à progressão clínica da aterosclerose em outros territórios.^3 , 10^ Tal fato decorre de possível aumento da carga de placa, o que resulta em maiores eventos conforme o índice FFR torna-se menor, como sugerido em estudos prévios.^14 - 16^ Nossos índices de ECAM foram um pouco piores do que aqueles encontrados no grupo FFR dos estudos FAME 2 e iFR- Swedeheart após seguimento de 5 anos, porém similares aos descritos no FAME 1, o que pode estar relacionado ao uso de *stents* farmacológicos de ultima geração nos dois primeiros estudos supracitados e também ao controle clínico mais rigoroso em pacientes recrutados para estudos clínicos randomizados.^10 , 13^

Os primeiros estudos com FFR demonstravam que valores baixos de FFR identificavam lesões com alta probabilidade de induzirem isquemia, e lesões com FFR < 0,8 não revascularizadas eram consideradas de alto risco.^9^ Acima desse valor, poderia se imaginar que pacientes com FFR normal-baixa (> 0,8-0,9) apresentariam maior risco de eventos causados pela progressão da doença aterosclerótica do que aqueles com valores normais-altos (> 0,9), o que foi demonstrado em estudos prévios, como o IRIS-FFR (Interventional Cardiology Research In-cooperation Society Fractional Flow Reserve), demonstrando maior ocorrência de desfechos nos pacientes com valor da FFR de 0,81-0,85, quando comparados ao grupo de FFR com valor ≥ 0,9. Contudo, nosso estudo diferiu do IRIS-FFR na estratificação dos valores de FFR, e não houve diferença nos desfechos para o grupo com FFR normal-baixa (0,81-0,9) quando comparado ao grupo com FFR normal-alta (> 0,9).^11^ Isso pode ter ocorrido pelo fato de os nossos intervalos terem sido amplos e o número de pacientes, limitado.

A determinação de isquemia por estenose coronariana pode ser considerada uma variável dicotômica, identificada por uma FFR acima ou abaixo de 0,8. Não foi feita, neste estudo, uma análise do *continuum* da FFR como foi realizado em alguns estudos,^11 , 17^ uma vez que este estudo não objetivava definir os valores de corte ideais para a FFR.

Nesse sentido, avaliamos pacientes com valores de FFR acima de 0,8, portanto sem evidência de isquemia, estratificados pelo grau de restrição ao fluxo coronariano em FFR normal-baixa (> 0,8-0,9) ou FFR normal-alta (> 0,9). Entretanto, nosso estudo não identificou diferença na incidência de desfechos clinicamente relevantes entre pacientes com diferentes estratos de FFR não isquêmico devido, possivelmente, aos intervalos de 0,81-0,9 e de > 0,9 serem muito amplos e ao número de pacientes ser limitado.

O conceito de FFR como marcador contínuo de risco foi estudado em uma metanálise com quase 6 mil pacientes, em que os autores encontraram que o melhor ponto de corte para revascularização seria < 0,75.^18^ No entanto, a relação entre o valor da FFR e os desfechos clínicos não foi detalhada entre os pacientes sem revascularização e com FFR > 0,8, e o acompanhamento clínico foi limitado a 1 ano e meio. Em outro estudo com acompanhamento de 2 anos,^16^ foi demonstrada maior ocorrência de ECAM conforme a diminuição do índice FFR, sugerindo novamente que, quanto menor o valor de FFR, maior o risco de ECAM em 2 anos.

O valor real da FFR prevalece sobre o valor prognóstico da gravidade da estenose angiográfica por estimativa visual, levando em consideração não apenas o segmento estenótico, mas também a carga aterosclerótica total do vaso e seu impacto na perfusão miocárdica regional. Quanto menor o valor da FFR, mais grave ou mais intensa será a isquemia para o mesmo nível de estresse.^19 , 20^

Em pacientes com síndrome coronariana aguda (SCA), a evolução pode estar amplamente associada à instabilidade clínica e à condição inflamatória sistêmica do paciente. Desta forma, não realizamos essa análise adicional, excluindo-se esse subgrupo. Além disso, há estudos indicando que o benefício da utilização da FFR em SCA é controverso.^14 , 21 , 22^

Neste trabalho, reportamos os desfechos clínicos dos pacientes em diferentes estratos de FFR com acompanhamento de até 5 anos. A incidência de eventos coronarianos é comparável às reportadas por outros estudos de acompanhamento a longo prazo com ou sem intervenção coronariana.^3 , 23 - 25^ Nosso estudo também apresenta achados comparáveis aos do estudo PROSPECT (A prospective Natural History Study of Coronary Atherosclerosis),^3^ em que a ocorrência do desfecho primário (morte, infarto ou re-hospitalização por angina instável ou angina progressiva) em 3 anos foi de 11,6% em lesões revascularizadas, porém não culpadas pela SCA.

O nosso estudo levanta a hipótese de que os pacientes com FFR normal-alta (> 0,9) não apresentam menor ocorrência de eventos quando comparados aos pacientes com FFR normal-baixa (> 0,8-0,9).

### Limitações do estudo

Quanto às limitações do estudo, tratou-se de uma análise observacional de dados armazenados em um banco de dados de um único centro de intervenção coronariana. O seguimento dos pacientes foi realizado através de consulta telefônica, o que pode ter impacto na avaliação dos desfechos a longo prazo. Foi realizada uma estratificação diferente de estudos previamente publicados devido ao tamanho reduzido da amostra. O tamanho da amostra deste registro e o número limitado de eventos podem ser insuficientes para revelar o real impacto clínico de acordo com a gravidade da lesão avaliada por FFR, o que levou à divisão dos grupos em estratos diferentes dos habitualmente utilizados nesse tipo de estudo. Para poder avaliar uma possível diferença entre os grupos FFR normal-baixa e FFR normal-alta, a amostra deveria ser próxima a 2 mil pacientes em cada grupo, o que tornaria o trabalho inviável em nosso meio. O período de seguimento de até 5 anos pode não ter sido suficiente para avaliação dos desfechos cardiovasculares pretendidos.

## Conclusão

Nosso estudo mostrou que os indivíduos do grupo isquêmico apresentam piores desfechos quando comparados aos grupos não isquêmicos. Entre os grupos que apresentam valores de FFR considerados normal-baixos e normal-altos, não houve diferença significativa na incidência de eventos cardiovasculares. Contudo, esses pacientes não devem ser considerados de baixo risco para eventos cardiovasculares a longo prazo, tendo em vista a possível progressão da placa aterosclerótica já demostrada em estudos prévios. Há necessidade de estudos prospectivos de longo prazo e com grande número de pacientes para melhor avaliar os desfechos cardiovasculares em pacientes portadores de estenose coronariana moderada com valores de FFR entre 0,8 e 1,0.
