# Supplementary material for: Avaliação Prognóstica da Reserva de Fluxo Fracionada em Diferentes Estratos nos Pacientes com Doença Arterial Coronariana
Source: Arq Bras Cardiol. 2023 Jun 7;120(6):e20211051. [Article in Portuguese] doi: 10.36660/abc.20211051 (PMC10263408; doi:10.36660/abc.20211051)
Supplement: Translation

# Translation: Prognostic Assessment of Fractional Flow Reserve in Different Strata in Patients with Coronary Artery Disease

**Authors:** Denise Pellegrini, Paulo R. A. Caramori, Ricardo Czarnobai Soccol, Ricardo Lasevitch, Gustavo Luís Agostini, Alice Dussin, Flavio Vinicius Costa Ferreira, Mario Bernardes Wagner, Luiz Carlos Bodanese

**Affiliations:** 1 Brazil Pontifícia Universidade Católica do Rio Grande do Sul, Porto Alegre, RS – Brazil; 2 Brazil Hospital São Lucas da PUCRS, Porto Alegre, RS – Brazil

**Keywords:** Fractional Flow Reserve, Outcomes, Ischemia

## Abstract

**Background:**

There are limited real-world data on the clinical course of untreated coronary lesions according to their functional severity.

**Objective:**

To evaluate the 5-year clinical outcomes of patients with revascularized lesions with fractional flow reserve (FFR) ≤ 0.8 and patients with non-revascularized lesions with FFR > 0.8.

**Methods:**

The FFR assessment was performed in 218 patients followed for up to 5 years. Participants were classified based on FFR into ischemia group (≤ 0.8, intervention group, n = 55), low-normal FFR group (> 0.8-0.9, n = 91), and high-normal FFR group (> 0.9, n = 72). The primary endpoint was major adverse cardiac events (MACEs), a composite of death, myocardial infarction, and need for repeat revascularization. The significance level was set at 0.05; therefore, results with a p-value < 0.05 were considered statistically significant.

**Results:**

Most patients were male (62.8%) with a mean age of 64.1 years. Diabetes was present in 27%. On coronary angiography, the severity of stenosis was 62% in the ischemia group, 56.4% in the low-normal FFR group, and 54.3% in the high-normal FFR group (p<0.05). Mean follow-up was 3.5 years. The incidence of MACEs was 25.5%, 13.2%, and 11.1%, respectively (p=0.037). MACE incidence did not differ significantly between the low-normal and high-normal FFR groups.

**Conclusion:**

Patients with FFR indicative of ischemia had poorer outcomes than those in non-ischemia groups. There was no difference in the incidence of events between the low-normal and high-normal FFR groups. Long-term studies with a large sample size are needed to better assess cardiovascular outcomes in patients with moderate coronary stenosis with FFR values between 0.8 and 1.0.

**Central Illustration:** : Prognostic Assessment of Fractional Flow Reserve in Different Strata in Patients with Coronary Artery Disease

FFR: fractional flow reserve; OR: odds ratio; CI: confidence interval

## Introduction

Physiological assessment of coronary artery disease (CAD) has become one of the cornerstones of decision-making for myocardial revascularization.

The anatomic severity of coronary lesions is associated with adverse events.^2 - 4^ Fractional flow reserve (FFR) has emerged as a reference tool for assessing the functional severity of coronary lesions. The significance of FFR in the treatment of CAD has been highlighted in recent years by the observation that coronary revascularization according to the functional significance of the lesion is associated with improved long-term clinical outcomes.^4 - 7^ There are published data on the clinical results of non-revascularized coronary lesions according to functional severity,^8 , 9^ and clinical outcomes are believed to differ according to the different FFR strata. However, the studies lack uniformity in the stratification of FFR values and, therefore, the outcomes may be different. Furthermore, the clinical factors that are associated with adverse clinical outcomes in non-revascularized lesions also differ between studies. Describing the clinical course and identifying the prognostic factors of non-revascularized lesions are of great relevance in clinical practice. Therefore, this study aimed to 1) evaluate the 5-year clinical outcomes of untreated lesions according to functional severity; and 2) define factors associated with adverse outcomes in untreated lesions. Due to the progression of atherosclerotic disease, the risk of events gradually increases with decreasing FFR values.^10^ Thus, patients who are left unrevascularized with a “low” FFR (i.e., 0.80 to 0.90) could be at increased risk of ischemia or complications than those with higher values (> 0.90).

The present study aimed to evaluate the incidence of outcomes in patients with different FFR strata after a 5-year follow-up period.

## Materials and Methods

### Study design

Observational historical cohort study. Data were collected prospectively and stored in the database of the interventional cardiology unit.

### Study endpoints

1. Major adverse cardiac events (MACEs), a composite of death, myocardial infarction, and need for repeat revascularization;
2. Death;
3. Myocardial infarction;
4. Ischemic stroke;
5. Need for repeat revascularization;
6. Target-vessel revascularization.

### Study population

Patients referred to the Cardiovascular Intervention and Interventional Cardiology Unit from January 2013 to September 2018 who underwent FFR assessment of at least one coronary lesion with at least 50% stenosis.

### Inclusion criteria

1. Patients with an indication for functional assessment of coronary lesions;
2. Coronary lesion with a ≥ 50% diameter stenosis by visual estimation on angiography.

### Exclusion criteria

1. Left main coronary artery lesion;
2. Cardiogenic shock;
3. Previous coronary artery bypass grafting (CABG);
4. Extremely tortuous and/or calcified coronary arteries;
5. Life expectancy of less than 2 years;
6. Pregnancy.

### Study protocol

Angiographic and non-angiographic data were recorded.

#### Coronary angiography

Coronary angiography was performed with a 5F or 6F diagnostic catheter via femoral or radial access. Selective intracoronary administration of 200 mcg of nitroglycerin was performed immediately before angiography of both left and right coronary arteries.^5 , 6^ Multiple cineangiographic images of the left and right coronary arteries were obtained in the left or right anterior oblique view, and cranial or caudal angulation if necessary.^7 , 10^

#### Fractional flow reserve (FFR)

A 6F guide catheter was routinely used to selectively catheterize the coronary artery for FFR measurement, paying attention to achieve coaxial alignment with the coronary ostium to avoid pressure damping. A 0.014’ guidewire with pressure monitoring was calibrated at atmospheric pressure, advanced to the end of the guide catheter, and equalized with the aortic pressure (Pa) of the guide catheter. The guidewire was then advanced to the distal part of the vessel to record the distal coronary pressure (Pd), ensuring that the pressure sensor was located beyond the lesion to be assessed. FFR of the left and right coronary lesions was recorded during maximal hyperemia induced by intravenous adenosine via good caliber peripheral access at a dose of 140 µg/kg/min for 3-5 minutes.^1 , 8 , 11^

FFR was automatically calculated as the lowest pressure gradient (Pa/Pd) reached during maximal hyperemia. An FFR of 0.8 or less was defined as hemodynamically significant (i.e., abnormal) according to current evidence, and revascularization was therefore indicated.^8 , 9 , 12^ Patients with lesions with an FFR > 0.8 or more were followed up clinically.

Participants were classified based on FFR into ischemia group (≤ 0.8, intervention group, n = 55), low-normal FFR group (> 0.8-0.9, n = 91), and high-normal FFR group (> 0.9, n = 72). Patients in the ischemia group were revascularized with drug-eluting stents.

#### Angiographic parameters

Quantitative coronary angiography (QCA) analysis of the lesion that was functionally assessed was performed using Cardiac Viewer (Philips™). Patients with at least 50% coronary stenosis (by visual assessment) in at least 2 vessels were considered to have multivessel disease.

#### Follow-up and clinical outcomes

Patients were followed for up to 5 years after the procedure. Follow-up was performed prospectively via telephone contact. The following clinical outcomes were assessed: death, acute myocardial infarction, repeat target-vessel revascularization, rehospitalization, and stroke. Target-vessel revascularization was defined as any percutaneous or surgical revascularization in a previously treated vessel due to restenosis or other lesion-related complication.

## Sample size

To detect a difference in MACEs of 12% in the low-normal FFR (0.8-0.9) and high-normal FFR (> 0.9) groups and of 30% in the ischemia group, at a ratio of 3:1, with a statistical power of 80% and a two-tailed significance level of 5%, a total sample size of 200 patients was necessary: 150 patients in the low-normal and high-normal FFR groups and 50 in the ischemia group.

## Statistical analysis

Quantitative data were expressed as mean (SD). Based on the central limit theorem, statistical tests were not used to assess the normality of quantitative data distribution. Categorical data were expressed as counts and percentages. One-way ANOVA was used to compare mean values, followed by Tukey’s post hoc test if necessary. The chi-square test or Fisher’s exact test was used to compare categorical events, followed by Benjamini-Hochberg correction if necessary. Odds ratio (OR) estimates and their respective 95% confidence intervals (CIs) were obtained in the univariate analysis with significance based on the chi-square test. Multivariate logistic regression models were used to calculate ORs adjusted for potential confounding effects. The significance level (two-tailed type I error) in this study was set at 0.05; therefore, results with a p-value < 0.05 were considered statistically significant. All analyses were performed using IBM-SPSS, version 25.0.

## Results

Participants were divided according to the FFR value into ischemia group (FFR ≤ 0.80, or intervention, n = 55), low-normal FFR group (> 0.8-0.9, non-revascularized, n = 91), and high-normal FFR group (> 0.9, non-revascularized, n = 72). A total of 241 patients were included in the study from January 2013 to September 2018, 218 of whom completed follow-up (90.4%). Mean follow-up was 3.5 years. Mean patient age was 64.1 years. Most patients were male (62.8%), and diabetes mellitus was present in 27% of patients. Other clinical characteristics are shown in Table 1 . Most baseline clinical characteristics did not differ between the groups, except for clinical presentation ( Table 1 ).

**Table 1:** – Demographic data

Clinical characteristics	Ischemia group n=55	Low-normal FFR group n=81	High-normal FFR group n=53	p value
Age (years)	63.2 ± 10.3	65.1± 10.4	64.2 ± 10.2	0.67
Male	37 (67.3)	58 (63.7)	42 (58.3)	0.57
Hypertension, n (%)	39 (70.9)	51 (56.0)	49 (68.1)	0.12
Dyslipidemia, n (%)	26 (47.3)	35 (38.5)	36 (50.0)	0.30
Current smokers, n (%)	14 (27.0)	20 (21.9)	23 (31.9)	0.47
Diabetes, n (%)	17 (30.9)	23 (25.3)	19 (25.0)	0.70
Previous myocardial infarction, n (%)	12 (21.8)	10 (11.0)	14 (19.4)	0.16
Previous coronary angioplasty, n (%)	21 (38.2)	25 (27.5)	24 (33.3)	0.24
Previous CABG, n (%)	1 (1.8)	5 (5.5)	6 (8.3)	0.28
**Clinical presentation, (n (%)**				
Chronic angina	41 (74.5)	81 (89.0)	53 (73.6)	0.02
Unstable angina	9 (16.4)	7 (7.7)	10 (13.9)	0.24
Non-ST segment elevation myocardial infarction	5 (9.1)	3 (3.3)	8 (11.1)	0.13
ST segment elevation myocardial infarction	0	0	1 (1.4)	0.36
**Medications, n (%)**				
ASA	43 (78.2)	67 (73.6)	52 (74.3)	0.64
Clopidogrel	30 (54.5)	49 (53.8)	36 (51.4)	0.72
Statin	42 (76.4)	71 (78.0)	52 (74.3)	0.75
Beta-blocker	33 (60.0)	56 (61.5)	39 (55.7)	0.59
Diuretic	12 (21.8)	22 (24.2)	20 (28.6)	0.39
ACEI	8 (14.5)	19 (20.9)	22 (31.4)	0.07
ARB	15 (23.7)	27 (29.7)	18 (25.7)	0.75
Oral hypoglycemic agent	15 (27.3)	17 (18.7)	17 (24.3)	0.76

*CABG: coronary artery bypass grafting; ASA: acetylsalicylic acid; ACEI: angiotensin-converting enzyme inhibitor; ARB: angiotensin receptor blocker*

Regarding angiographic characteristics, the most frequently assessed vessel was the left anterior descending coronary artery in the ischemia group, followed by the low-normal FFR group ( Table 2 ). The degree of angiographic stenosis also differed between the groups, being more severe in the ischemia group ( Table 2 ).

**Table 2:** – Angiographic data

Angiographic data	Ischemia group n=55	Low-normal FFR group n=91	High-normal FFR group n=72	p value
Vessel assessed				<0.001
Anterior descending coronary artery, n (%)	47 (85.5)	60 (65.9)	31 (43.1)	*
Vessels other than the anterior descending artery, n (%)	8 (14.5)	31 (34.1)	41 (56.9)	
Angiographic stenosis, n (%)	62.0 ± 7.7	56.5 ± 6.2	54.0 ± 8.3	<0.001
FFR	0.75 ± 0.03	0.85 ± 0.02	0.94 ± 0.03	<0.001

* p ≤ 0.001 for frequency of assessment of the anterior descending coronary artery between the low-normal and high-normal FFR groups. FFR: fractional flow reserve.

The primary endpoint (MACEs), a composite of death, myocardial infarction, need for repeat revascularization, rehospitalization, and stroke, differed significantly between the groups, occurring in 25.5% in the ischemia group, 13.2% in the low-normal FFR group, and 11.1% in the high-normal FFR group (p = 0.037) ( Table 3 ). The OR for the occurrence of MACEs from the ischemia group to the high-normal FFR group was 2.73 (95% CI 1.03-7.09; p = 0.039). The OR for the occurrence of MACEs from the ischemia group to the low-normal FFR group was 2.25 (95% CI 0.95-5.29; p = 0.064), and the OR for the occurrence of MACEs from the low-normal FFR group to the high-normal FFR group was 1.22 (95% CI 0.47-3.15; p = 0.680) ( Figure 1 ).

**Table 3:** – Outcomes

Outcomes, n (%)	Ischemia group n=55	Low-normal FFR group n=91	High-normal FFR group n=72	p value
Composite outcome	14 (25.5)	12 (13.2)	8 (11.1)	0.037
Death	2 (3.6)	6 (6.6)	3 (4.2)	0.67
Acute myocardial infarction	1 (1.8)	3 (3.3)	1 (1.4)	0.70
Stroke	1 (1.8)	0 (0.0)	1 (1.4)	0.46
Need for repeat angioplasty	12 (21.8)	5 (5.5)	5 (7.1)	0.04
Target-vessel revascularization	6 (10.9)	3 (3.3)	3 (4.3)	0.12
Need for rehospitalization	12 (23.6)	6 (6.6)	6 (8.6)	0.05
Need for cardiac catheterization	15 (27.3)	7 (7.7)	10 (14.3)	0.05

**Figure 1:** – Between-group comparison of MACEs. FFR: fractional flow reserve.

The mortality rate was 3.6% in the ischemia group, 6.6% in the low-normal FFR group, and 4.2% in the high-normal FFR group (p = 0.67). Myocardial infarction occurred in 1 patient in the ischemia group, in 3 patients in the low-normal FFR group, and in 1 patient in the high-normal FFR group (p = 0.70). A significantly higher rate of patients underwent repeat angioplasty during clinical follow-up in the ischemia group (21.8%) than in the low-normal (5.5%) and high-normal (7.1%) FFR groups (p = 0.04) ( Table 3 ). Stroke occurred in 1 patient in the ischemia and in the high-normal FFR group. No stroke occurred in the low-normal FFR group. The need for rehospitalization was significantly higher in the ischemia group (23.6%) than in the low-normal (6.6%) and high-normal (8.6%) FFR groups (p = 0.01).

A subgroup analysis evaluating patients with chronic CAD, who account for most participants in the study (175 of 218 patients, 80.2%), revealed a significant difference in the occurrence of MACEs between the groups. The MACE rate was 29.3% in patients with FFR ≤ 0.8, 11.1% in the low-normal FFR stratum, and 7.5% in the high-normal FFR stratum ( Table 4 ). The difference in the occurrence of MACEs was probably due to the greater need for repeat revascularization in the ischemia group. There was no significant difference in the occurrence of the outcomes between the low-normal and high-normal FFR groups (p = 0.56).

**Table 4:** – Outcome in the subgroup of patients with chronic coronary artery disease

Outcomes, n (%)	Ischemia group n=41	Low-normal FFR group n=81	High-normal FFR group n=53	p value
Composite outcome	12 (29.3)	9 (11.1)	4 (7.5)	0.004
Death	0	0	2 (3.8)	0.07
Acute myocardial infarction	1 (2.4)	2 (2.5)	0	0.34
Stroke	1 (2.4)	0	1 (2.0)	0.91
Need for repeat angioplasty	10 (24.4)	4 (4.9)	1 (2.0)	<0.001
Target-vessel revascularization	2 (1.1)	1 (2.4)	1 (2.0)	0.26
Need for rehospitalization	11 (26.8)	5 (6.2)	3 (5.9)	0.003
Need for cardiac catheterization	11 (26.8)	6 (7.4)	5 (9.8)	0.02

A univariate risk factor analysis was performed to identify MACE predictors in all participants and showed that being male, age > 65 years, hypertension, diabetes mellitus, dyslipidemia, previous myocardial infarction, previous angioplasty, and previous CABG were not significantly associated with major cardiovascular events analyzed separately. However, FFR ≤ 0.80 was a predictor of MACEs (OR 2.73, 95% CI 1.05-7.09; p = 0.039) ( Table 5 ).

**Table 5:** – Univariate analysis for MACE predictors

Variable	Without MACE n = 184	With MACE n = 34	OR	95% CI	p value
Male, n **(** %)	116 (62.7)	22 (64.7)	0.91	(0.42-1.96)	0.82
Age > 60 years, n **(** %)	123 (66.5)	22 (64.7)	0.92	(0.42-1.98)	0.84
FFR, n/total, n **(** %)					
≥ 0.90	64/184 (34.8)	8/34 (23.5)	1	-	-
0.81-0.89	79/184 (42.9)	12/34 (35.3)	1.22	(0.47-3.15)	0.689
≤ 0.80	41/184 (22.3)	14/34 (41.2)	2.73	(1.05-7.09)	0.039
Hypertension, n (%)	120 (64.9)	20 (58.8)	0.77	(0.37-1.63)	0.561
Diabetes mellitus, n (%)	46 (24.9)	12 (35)	1.64	(0.75-3.5)	0.20
Dyslipidemia, n (%)	83 (44.9)	14 (41.2)	0.86	(0.41-1.80)	0.69
Previous myocardial infarction, n (%)	30 (16.2)	6 (17.6)	1.1	(0.42-2.90)	0.83
Previous angioplasty, n (%)	10 (5.4)	2 (5.9)	1.09	(0.2-5.22)	0.91
Previous CABG, n (%)	57 (30.8)	12 (38.2)	1.39	(0.65-2.96)	0.39

MACE: major adverse cardiac event; OR: odds ratio; FFR: fractional flow reserve; CABG: coronary artery bypass grafting.

In the multivariate analysis, after adjusting for sex, age, hypertension, and diabetes mellitus, the difference in MACE occurrence between the FFR strata remained unchanged (OR 2.72, 95% CI 1.03-7.14; p = 0.04).

## Discussion

The present study, involving 218 patients with CAD followed for up to 5 years and subjected to FFR assessment, showed a larger number of MACEs in the ischemia group than in the low-normal and high-normal FFR groups, with no differences between the latter two groups.

Anatomic severity assessment parameters, such as diameter stenosis, extent, plaque eccentricity, angle and calcification, may be indicative of the complexity and prognosis of the lesion. However, the prognosis differs significantly between patients depending on whether or not myocardial ischemia is present in a given lesion.^5^ Therefore, there is a need to overcome the limitations of angiography in the assessment of the functional impact of coronary lesions, for which the FFR has been used as a reference tool in invasive physiological assessments.^6^

In recent years, several studies have consistently reported the favorable results of the FFR-guided revascularization strategy in clinical practice. In the DEFER trial, clinical outcomes following non-intervention on the basis of FFR measurement were excellent for a 15-year follow-up.^8^ In the Fractional Flow Reserve versus Angiography for Multivessel Evaluation (FAME) 1 trial, routine measurement of FFR was also shown to significantly reduce the rate of the composite clinical endpoint of death, nonfatal myocardial infarction, and repeat revascularization in patients with multivessel CAD who were treated with drug-eluting stents.^10^ Furthermore, the FAME 2 trial showed that FFR-guided angioplasty plus optimal medical therapy decreased the need for urgent revascularization compared with optimal medical therapy alone in stable patients with functionally significant coronary lesions.^13^ Considering the results of these trials, the treatment of coronary lesions guided by FFR measurements can ensure better clinical outcomes.

In the present study, the long-term prognosis of patients with revascularized ischemic coronary lesions was significantly worse than that of patients with non-revascularized non-ischemic coronary lesions. This indicates that, in the real world, revascularization with drug-eluting stents associated with conventional clinical management was unable to reduce outcomes in patients with ischemia to the point that they become comparable to the outcomes of patients without ischemia as determined by FFR.

The higher MACE rate in the ischemia group was due to the greater need for repeat revascularization mainly of other lesions. This finding indicates that patients with ischemic lesions have worse clinical outcome related to the clinical progression of atherosclerosis in other territories.^3 , 10^ This results from a possible increase in plaque burden that leads to an increase in the risk of events as the FFR values decrease, as suggested in previous studies.^14 - 16^ Our MACE rates were slightly worse than those found for the FFR group in the FAME 2 and iFR-SWEDEHEART trials after 5-year follow-up, but similar to those reported in the FAME 1 trial, which may be related to the use of new-generation drug-eluting stents in the first two aforementioned studies and also to a more rigorous clinical management in patients recruited for randomized controlled trials.^10 , 13^

The first studies with FFR demonstrated that low FFR values could identify lesions with high potential to induce ischemia, and non-revascularized lesions with an FFR < 0.8 were at increased risk.^9^ Above this value, it was assumed that patients with low-normal FFR (> 0.8-0.9) would be at greater risk of events caused by the progression of atherosclerotic disease than those with high-normal FFR (> 0.9), which was demonstrated in previous studies such as the Interventional Cardiology Research In-cooperation Society Fractional Flow Reserve (IRIS-FFR) registry, in which a higher rate of outcomes was reported in patients with an FFR of 0.81-0.85 than in those with an FFR ≥ 0.9. However, our study differed from the IRIS-FFR registry in the stratification of FFR values and showed no difference in the outcomes between the low-normal (0.81-0.9) and high-normal (> 0.9) FFR groups.^11^ This may have resulted from our wide range of values and limited number of participants.

Ischemia determined by coronary stenosis can be considered a dichotomous variable, identified by an FFR above or below 0.8. We did not perform a continuum analysis of FFR as performed in previous studies,^11 , 17^ because we did not aim to define the optimal cutoff value for FFR.

In the present study, we evaluated patients with FFR values > 0.8 (i.e., without evidence of ischemia) stratified by the degree of coronary flow restriction into low-normal FFR (> 0.8-0.9) and high-normal FFR (> 0.9). However, we found no difference in the incidence of clinically relevant outcomes between patients with different strata of non-ischemic FFR possibly because the range of values (0.81-0.9 and > 0.9) was too wide and the number of patients was limited.

The concept of FFR as a continuous risk marker was investigated in a meta-analysis of nearly 6000 patients, which found that the optimal cutoff point for revascularization would be < 0.75.^18^ However, the relationship of FFR values with clinical outcomes was not detailed between patients without revascularization with an FFR > 0.8, and clinical follow-up was limited to 1.5 years. Another study with a 2-year^16^ follow-up demonstrated that MACEs increased as FFR decreased, again suggesting that lower FFR values increase the risk of MACEs at 2 years.

The actual FFR value prevails over the prognostic value of the severity of the angiographic stenosis by visual estimation, taking into account not only the stenotic segment but also the total coronary atherosclerotic burden and its impact on regional myocardial perfusion. For the same stress level, ischemia increases in severity or intensity with decreasing FFR values.^19 , 20^

In patients with acute coronary syndrome, the outcome may be largely associated with clinical instability and the patient’s systemic inflammatory condition. Therefore, we did not perform an additional analysis excluding this subgroup. Furthermore, there are studies indicating that the benefit of using FFR in acute coronary syndromes is controversial.^14 , 21 , 22^

In the present study, we reported the clinical outcomes of patients with different FFR strata after a follow-up of up to 5 years. The incidence of coronary events was comparable to that reported by previous long-term follow-up studies with or without coronary intervention.^3 , 23 - 25^ Our study also presents findings comparable to those of the PROSPECT study (A Prospective Natural-History Study of Coronary Atherosclerosis),^3^ in which the 3-year cumulative rate of the primary endpoint (death, myocardial infarction, or rehospitalization due to unstable or progressive angina) was 11.6% in revascularized, nonculprit coronary lesions.

Our study suggests the hypothesis that patients with high-normal FFR (> 0.9) do not have a lower rate of events than patients with low-normal FFR (> 0.8-0.9).

### Study limitations

Limitations of the study include the fact that it is an observational analysis of data stored in a database of a single coronary intervention center. Patient follow-up was performed via telephone contact, which may have had an impact on the assessment of long-term outcomes. Stratification differed from that of previous publications because of our small sample size. The number of patients and events in our study could be insufficient to reveal the real clinical impact according to lesion severity assessed by FFR, which led us to divide the groups into FFR strata different from those usually used in this type of study. To detect a possible difference between the low-normal and high-normal FFR groups, the sample size should be close to 2000 patients per group, which would render the study unfeasible in our setting. The follow-up period of up to 5 years may not have been sufficient to assess the intended cardiovascular outcomes.

## Conclusion

Our results showed that patients in the ischemia group had poorer outcomes than those in the non-ischemia groups. There was no significant difference in the incidence of cardiovascular events between the low-normal and high-normal FFR groups. However, these patients should not be considered at low risk for long-term cardiovascular events given the possible progression of atherosclerotic plaque already demonstrated in previous studies. Long-term prospective studies with a large sample size are needed to better assess cardiovascular outcomes in patients with moderate coronary stenosis with FFR values between 0.8 and 1.0.
